# Scanning Probe Microscopy on heterogeneous CaCu_3_Ti_4_O_12 _thin films

**DOI:** 10.1186/1556-276X-6-118

**Published:** 2011-02-04

**Authors:** Patrick Fiorenza, Raffaella Lo Nigro, Vito Raineri

**Affiliations:** 1 Istituto per la Microelettronica e Microsistemi (IMM), Consiglio Nazionale delle Ricerche, Strada VIII, 5; 95121 Catania, Italy

## Abstract

The conductive atomic force microscopy provided a local characterization of the dielectric heterogeneities in CaCu_3_Ti_4_O_12 _(CCTO) thin films deposited by MOCVD on IrO_2 _bottom electrode. In particular, both techniques have been employed to clarify the role of the inter- and sub-granular features in terms of conductive and insulating regions. The microstructure and the dielectric properties of CCTO thin films have been studied and the evidence of internal barriers in CCTO thin films has been provided. The role of internal barriers and the possible explanation for the extrinsic origin of the giant dielectric response in CCTO has been evaluated.

## I. Introduction

The electrical properties of CaCu_3_Ti_4_O_12 _(CCTO) ceramics and single crystals received considerable attention due to the effective huge permittivity (up to 10^5^) measured in the radio frequencies range, furthermore stable in the 100-400 K temperature range [1-3]. In the recent literature, this giant permittivity has been commonly related to extrinsic effects, i.e. not associated to the bulk material property itself. Possible extrinsic mechanisms to account for the colossal permittivity behaviour have been supported by results from impedance spectroscopy (IS) [4], Raman spectroscopy [5] and first-principles calculations [6]. In particular, the IS data on CCTO polycrystalline ceramics reported so far, have been modelled considering an equivalent circuit of two elements, each consisting of a parallel resistor-capacitor (RC), connected in series. One RC element (R_gb _and C_gb_) simulates the grain boundary response, whereas the other (R_b _and C_b_) simulates the bulk contribution [4]. The model is suitable to simulate, in a first approximation, the measured capacitance (C) vs. frequency (f) curves showing relaxation at high frequencies. Therefore, the origin of the huge permittivity, arising from the capacitive response before the observed relaxation, has been mainly attributed to an internal barrier layer capacitor (IBLC) behaviour associated with insulating grain boundaries and semiconducting grains structure. This explanation has been corroborated imaging the insulating barriers at the grain boundaries of CCTO ceramics by both nanocontact current-voltage measurements [7] and Scanning Probe Microscopy (SPM) with conductive tips [8,9] as already demonstrated on other microelectronic investigation [10,11].

However, for microelectronics applications, CCTO thin films are much more interesting than ceramics, thus for those applications the occurrence and the origin of the high permittivity deserve to be reliable demonstrated and studied specifically in thin films. In this context, it should be noted that the IBLC model cannot be responsible for the giant permittivity observed in CCTO single crystals [12] as well as in epitaxial columnar thin films [13], where no grain boundary is crossed between the two planar electrodes parallel to the surface. In fact, the giant response, indeed observed nowadays in thin films, has been explained considering an electrode effect according to the Maxwell-Wagner (MW) model [14], and this raises the question, to date not definitively studied and discussed, about the CCTO capacitor reliability and the importance of Schottky barriers at the electrode-surface interfaces [15].

In this paper, we report on CCTO thin films deposited by Metal-Organic Chemical Vapor Deposition (MOCVD) possessing a "bricks wall" (BW) morphology and a giant permittivity. In this case the IBLC effect can be present. Here, we demonstrate its occurrence and we evaluate the necessary conditions for a reproducible achievement of huge capacitive density in CCTO integrated condensers.

## II. Experimental

CCTO films have been deposited by a two-steps MOCVD processes on IrO_2_/Ir/TiO_2_/SiO_2_/Si substrate using the condition parameters described elsewhere and 180 minutes deposition time [16-18].

The electrical characterization at nanometre scale was performed by a VEECO D3100 atomic force microscope (AFM) equipped with a Nanoscope V controller and the Nanoman head operating in air, in contact mode and in closed loop condition, using the Conductive Atomic Force Microscopy (C-AFM) module. Standard experiments were carried out using Nanoworld boron doped diamond tips [19-22]. Laser off measurements have been also carried out to exclude the influence of the laser on the reported electrical measurements at nanoscale.

The macroscopic capacitances versus frequency (C-f) measurements were carried out on Pt/CCTO/IrO_2 _capacitors by adopting the Terman method and using a HP 4284A equipment at an *AC *voltage with a fixed amplitude of 50 mV. The test devices have been fabricated with top electrodes having an area of 10^4 ^μm^2 ^obtained by a photolithographic lift-off process of the sputtered platinum layer.

The macroscopic characteristics were collected at different temperatures, in a range from 298 to 473 K.

## III. Results

**Figure 1:**
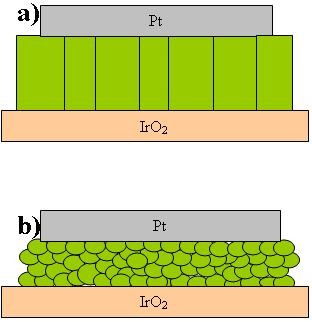
**Schematic cross section of CCTO thin films possessing columnar (a) and "bricks wall" like (b) morphologies**.

Several papers reported on CCTO thin films grown by PLD (Pulsed Laser Deposition) or others physical methodologies presenting columnar morphologies (Figure 1a) where no barriers parallel to the electrodes are present similarly to single crystal [23,24]. Our CCTO thin films have been grown on IrO_2_/Ir/TiO_2_/SiO_2_/Si substrate by MOCVD, a more industrial friendly technique. They are polycrystalline with rounded grains about 100 nm wide. The film morphology is similar to that observed in ceramics, called "bricks wall" (BW) morphology, and is characterized by many grain boundaries parallel to the electrode surface (Figure 1b) in contrast with the typical columnar growth (Figure 1a) observed in CCTO films deposited by PLD.

**Figure 2:**
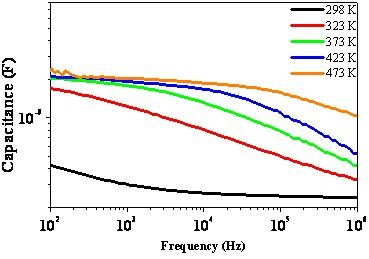
**C-f curves at different temperatures on the as-fabricated Pt/CCTO/IrO_2 _capacitors**.

Capacitance vs. frequency (C-f) curves have been measured in the 10^2^-10^6 ^Hz range and at different temperatures from 298 up to 473 K. Typical capacitance versus frequency curves (Figure 2) have been collected at several temperatures and both point out to a peculiar temperature dependent relaxation behaviour: the relaxation frequency increases upon increasing temperature. This trend, observed by macroscopic measurements, is similar to that found in CCTO ceramics, thus it could be also interesting the comparison of the dielectric behaviours at nanoscale.

**Figure 3:**
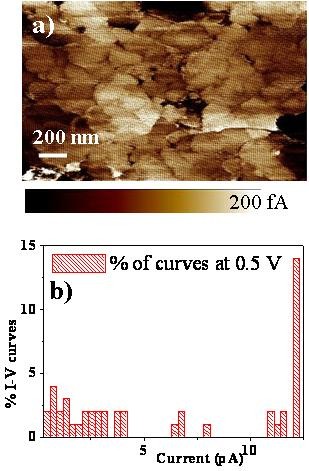
C-AFM current map (a) collected on CCTO thin films, I-Vs acquired in a 10 × 10 matrix and its distribution histogram

The nanoscale mapping of the electrical response is reported in Figure 3 at room temperature. It was carried out in order to distinguish the presence of an internal barrier [25] or a superficial polarization [26]. The current map (a) has been collected on the bare CCTO thin film surface. Insulating grain boundaries and conducting grains are clearly visible (Figure 3a). This dielectric structure recalls the CCTO ceramics considering also the BW morphology. Further details have been provided by the current versus voltage (I-V) curves, locally collected by C-AFM on a 10x10 matrix points, each spaced of 200 nm. The I-V curves clearly belong to two families as reported in the related histogram (Figure 3b). The first family is centred at high current values and the second at quite lower current values. They can be respectively related to the current flowing through the grain (when the tip is statistically contacting a grain) or the grain boundaries (when the tip is occasionally contacting the grain boundaries). The current flowing through the grain boundaries is at least two orders of magnitude lower than in the grains as already observed in CCTO polycrystalline ceramics [27].

The present CCTO films possess a BW structure with conducting grains surrounded by insulating grain boundaries, thus prompting to consider the IBLC model as a possible explanation for the observed temperature dependence of the relaxation frequencies.

## IV. Discussion

Previous reports [26,27] have shown that the microstructure and the electrical properties of CCTO ceramics are strongly dependent on processing conditions. In fact, the grain size increases with increasing the sintering temperature and/or the processing time as well [26,27]. The presence of the IBLC effect on CCTO ceramics has been also reported and related to the synthesis conditions.

The fabrication of "bricks wall" CCTO thin films encourages the analogy with the ceramics (not possible for columnar films). Both the presence of a temperature relaxation frequency dependence (Figure 2a) and the presence of insulating grain boundaries surrounding semiconducting grains (Figure 3a) urges the use of the IBLC model to explain the giant permittivity response in thin films.

Considering now the dielectric characteristics (Figure 2) when the IBLC is present, the temperature dependent relaxation frequency can be used to study the electrical properties of the grain boundaries. Their barrier height can be determined by measuring the current flowing in a wide temperature range (298-473 K). In fact, the presence of internal barriers can be related to a hopping transport model inducing a thermal activated conductivity [7]. The Arrhenius plot of the measured conductivity allowed to estimate the grain boundary barrier activation energy, it is E_a_~0.25 eV. This measured activation energy for the conduction in the CCTO films is lower than found in ceramics [26,27]; this discrepancy can be essentially explained by the different conducting/insulator volume fraction in the two cases due mainly to the huge difference in the grain size.

Finally, it is noteworthy that remarkable high capacitance density (about 100 nF/mm^2^) can be achieved at room temperature with a reasonable dispersion factor (tanδ < 1 at 1 MHz) and in a wide frequency range (10^2^-10^6 ^Hz) at 473 K.

## V. Conclusion

CCTO thin films presenting a BW structure have been fabricated by MOCVD. In these films the main mechanism has been proposed for the explanation of the extrinsic giant permittivity response. The presence of the IBLC effect was demonstrated. Remarkable high capacitance density (about 100 nF/mm^2^) can be achieved at room temperature.

## Competing interests

The authors declare that they have no competing interests.

## Authors' contributions

PF carried out the electrical characterization and conceived of the study. RL performed the film deposition and conceived of the study. VR conceived of the study and participated in its design and coordination section. All authors read and approved the final manuscript.
